# Application of optimized Kalman filtering in target tracking based on improved Gray Wolf algorithm

**DOI:** 10.1038/s41598-024-59610-6

**Published:** 2024-04-18

**Authors:** Zheming Pang, Yajun Wang, Fang Yang

**Affiliations:** Department of Electronic and Information Engineering, Liaoning University of Technology, Jinzhou, 121001 China

**Keywords:** Improved Gray Wolf algorithm, Optomized Kalman filter, Nonlinear control parameters, Target tracking, Natural hazards, Electrical and electronic engineering

## Abstract

High precision is a very important index in target tracking. In order to improve the prediction accuracy of target tracking, an optimized Kalman filter approach based on improved Gray Wolf algorithm (IGWO-OKF) is proposed in this paper. Since the convergence speed of traditional Gray Wolf algorithm is slow, meanwhile, the number of gray wolves and the choice of the maximum number of iterations has a great influence on the algorithm, a nonlinear control parameter combination adjustment strategy is proposed. An improved Grey Wolf Optimization algorithm (IGWO) is formed by determining the best combination of adjustment parameters through the fastest iteration speed of the algorithm. The improved Grey Wolf Optimization algorithm (IGWO) is formed, and the process noise covariance matrix and observation noise covariance matrix in Kalman filter are optimized by IGWO. The proposed approach is applied into. The experiment results show that the proposed IGWO-OKF approach has low error, high accuracy and good prediction effect.

## Introduction

Currently, trajectory prediction methods have made significant progress in the fields of computer vision and machine learning^1^. These methods include techniques based on physical models^2–5^, statistical models, machine learning^6^ and deep learning^7^. Physical and statistical models can utilize the physical properties of the target and historical trajectory data to make predictions, while machine learning and deep learning methods make predictions by learning the modal and temporal dependencies of large amounts of trajectory data. In addition, some studies have fused multimodal information into trajectory prediction to improve accuracy^8–12^. Although some results have been achieved, they still face challenges such as complex scenarios, multi-target interactions, and uncertainty, which require further research and improvement.

Trajectory prediction research in China started earlier, and the traditional methods mainly focus on the trajectory prediction methods based on techniques such as rules, statistics and machine learning. For example, trajectory prediction methods based on mathematical models such as Kalman filter and particle filter are widely used in the fields of target tracking and traffic management^13–15^. In recent years, domestic scholars began to adopt deep learning methods for trajectory prediction research. By using techniques such as convolutional neural network (CNN), recurrent neural network (RNN) and attention mechanism, the accuracy and robustness of trajectory prediction have been improved. Meanwhile, some trajectory prediction models for specific scenarios have also achieved good results, such as traffic flow prediction and pedestrian behavior prediction^16–18^. Foreign trajectory prediction research emphasizes the mining and analysis of large-scale real data. By collecting and utilizing a large amount of data such as mobile devices and traffic monitoring systems, researchers apply machine learning and deep learning methods for trajectory prediction. In addition, fusing other data sources such as geographic information and social networks has become a trend. They build relevant models based on factors such as historical behavioral patterns, environmental characteristics and social interactions of the target individuals and use them to predict future movement trajectories. These include modeling methods based on Markov Decision Process (MDP) and Markov Random Field (MRF), etc.

Kalman filtering algorithm is a classical method for trajectory prediction. Kalman filtering algorithm is characterized by recursive computation, which can efficiently handle real-time data streams and is suitable for application scenarios that require real-time updating of state estimates.It can effectively suppress the influence of measurement noise and improve the accuracy and precision of landslide monitoring. In addition, Kalman filter can also detect the possible occurrence of landslide in advance by predicting the future state change trend^19–21^. Lu put forward application of Kalman filter model in the landslide deformation forecast^22^. Li put forward research on prediction of metro surface deformation based on ensemble Kalman filter^23^. However, it suffers from deficiencies such as fixed filtering parameters, so it is proposed to optimize Kalman filtering with an improved Gray Wolf algorithm.

## Improving the Gray Wolf optimization algorithm

Grey Wolf Optimization (GWO) is an optimization algorithm inspired by the social behavior of grey wolves. The algorithm has some significant advantages and some potential disadvantages. First, one of the advantages of Grey Wolf Optimization algorithm is its simplicity and ease of implementation. The principles of the algorithm are relatively simple and easy to understand and implement. Second, the Gray Wolf Optimization algorithm has a strong global search capability. Inspired by the prey-seeking behavior of grey wolf packs, the algorithm is able to effectively explore potential optimal solutions in the entire search space. This makes it perform well in unconstrained continuous optimization problems and is able to find globally optimal solutions. In addition, the Gray Wolf Optimization algorithm typically has a fast convergence rate. By simulating the collaborative and competitive behavior of wolves to update the solution vector, the algorithm is able to quickly converge to a better solution, thus speeding up the optimization process. Finally, the Gray Wolf Optimization algorithm has some parameter adaptivity. It can automatically adjust the parameter values in the search process to improve the robustness and performance of the algorithm. This adaptability can adapt to the characteristics of different problems and reduce the difficulty of parameter adjustment. However, the Gray Wolf Optimization algorithm also has some potential drawbacks. First, it is more sensitive to the constraints of the problem. When dealing with constrained optimization problems, additional processing is required to ensure the feasibility of the results. Second, although the Gray Wolf Optimization algorithm has good global search capability, it may still fall into local optimal solutions in some complex problems. Finally, parameter tuning in the algorithm is relatively difficult; different problems may require different parameter settings, and experimentation and debugging are needed to obtain better performance.

Taking $\alpha$ as the optimal solution (individual's fitness is optimal), the second best solution $\beta$, the best solution $\delta$, and the remaining candidate solution named $\omega$. The hunting process is guided by $\alpha \beta \delta$ and $\omega$ follows these three wolves. That is, we always go to the three best solutions and then search around the region with the aim of finding better solutions and then updating $\alpha \beta \delta$.

During the hunting process, the behavior of the gray wolves rounding up their prey is defined as follows:

Formula for the distance between an individual and a hunt:

1 $$D = \left| {C \cdot X_{P} (t) - X(t)} \right|$$

Gray Wolf location update formula:

2 $$X\left( {t + 1} \right) = X_{P} \left( t \right) - A \cdot D$$

coefficient vector:

3 $$A = 2a \cdot r_{1} - a$$

4 $$C = 2 \cdot r_{2}$$

where t is the number of iterations, D is a vector of distances between individuals and hunts, '$\cdot$' is not a dot product, it is a multiplication, $X_{P}$ is a vector of hunts' locations, X is a vector of Gray Wolf locations, $a$ is a control parameter (decreasing linearly from 2 to 0 with the number of iterations), and r1 and r2 are random vectors, modulo a random number between (0 and 1).

From the formula, it can be seen that after moving the Gray Wolf pack to $\alpha$, the direction of movement is determined by its own position and the random vector C, and the movement step length is determined by the isolation distance from the Gray Wolf distance and the coefficient vector A, i.e., $\alpha$ linear decrease implies the randomness and the magnitude of the movement step length, and the step length decreases with the number of iterations getting closer and closer to the optimal solution.

The mathematical model of individual Gray Wolf tracking prey location is described as follows:

5 $$\left\{ {\begin{array}{*{20}l} {D_{\alpha } = {\text{|C}}_{{1}} \cdot {\text{X}}_{\alpha } - {\text{X|}}} \hfill \\ {D_{\beta } = {\text{|C}}_{{2}} \cdot {\text{X}}_{\beta } - {\text{X|}}} \hfill \\ {D_{\delta } = {\text{|C}}_{{3}} \cdot {\text{X}}_{\delta } - {\text{X|}}} \hfill \\ \end{array} } \right.$$

where $D_{\alpha }$, $D_{\beta }$ and $D_{\delta }$ denote the distances between $\alpha$ and $\beta$, $\delta$ and other individuals, respectively; $X_{\alpha } X_{\beta } X_{\delta }$ represents the current positions of $\alpha$ and $\beta$,$\delta$, respectively; $C_{1} C_{2} C_{3}$ is a random vector, and X is the current position of the Gray Wolf.

6 $$\left\{ {\begin{array}{*{20}l} {X_{1} = X_{\alpha } - A_{1} \cdot \left( {D_{\alpha } } \right)} \hfill \\ {X_{2} = X_{\beta } - A_{2} \cdot \left( {D_{\beta } } \right)} \hfill \\ {X_{3} = X_{\delta } - A_{3} \cdot \left( {D_{\delta } } \right)} \hfill \\ \end{array} } \right.$$

7 $$X_{t + 1} = \frac{{X_{1} + X_{2} + X_{3} }}{3}$$

Equation (6) defines the step length and direction of individual $\omega$ in the wolf pack toward $\alpha$ and $\beta$, $\delta$, respectively, and Eq. (7) defines the final position of $\omega$.dierji.

The improved Gray Wolf algorithm is formulated as follows:

8 $$a_{1} \left( t \right) = a_{first} - \left( {a_{first} - a_{final} } \right) \cdot \sin \left( {\frac{1}{\mu }\left( {\frac{t}{{T_{\max } }}} \right)^{\lambda } \cdot \pi } \right)$$

where $a_{first}$ and $a_{final}$ are the initial and final values of the control parameters, respectively; $\mu$ and $\lambda$ are the regulation parameters, and $T_{\max }$ is the maximum number of iterations. By adjusting the regulation parameters, the convergence speed of the local and global search of the Gray Wolf algorithm is thereby improved.

## Gray Wolf optimization algorithm for improved Kalman filtering

Kalman filtering has a wide range of applications in state estimation and optimization problems, but it also has some shortcomings. Meanwhile, combining the Gray Wolf algorithm with Kalman filtering can make up for these shortcomings and bring the following benefits: firstly, Kalman filtering performs poorly in dealing with nonlinear and non-Gaussian problems, and it is easy to fall into local optimal solutions. And the Gray Wolf algorithm, as a global optimization algorithm, can provide stronger global search capability. By combining the two, the global search ability of the Gray Wolf algorithm can be used to solve the limitations of Kalman filtering in nonlinear problems, making the optimization process more comprehensive and accurate. Second, Kalman filtering is highly sensitive to measurement noise and system modeling errors. These errors may cause Kalman filtering to fail to accurately estimate the system state, thus affecting the accuracy of the optimization results. The Gray Wolf algorithm, on the other hand, can reduce the dependence on a single objective through the diversity search strategy, thus improving the robustness and stability of the algorithm. Therefore, introducing the Gray Wolf algorithm into Kalman filtering can enhance the tolerance of the algorithm to measurement noise and model error, and improve the accuracy and reliability of the optimization results.

In summary, combining the Gray Wolf algorithm with Kalman filtering can give full play to the advantages of both and make up for their respective shortcomings. Through the global search capability and robustness of the Gray Wolf algorithm, combined with the state estimation and correction process of Kalman filtering, more accurate, stable and globally optimized results can be achieved, which is suitable for state estimation and optimization tasks in complex problems and real-time systems.

In the measurement update proposed to be solved iteratively thus optimizing the calculation of the filter gain, the efficiency of the filter estimation is affected due to the presence of ambient noise effects. Therefore, an adaptive forgetting factor is introduced to correct the one-step prediction covariance matrix in real time in order to correct the filter gain matrix and improve the algorithm's target tracking efficiency. Calculate the corrected one-step prediction covariance matrix:

9 $$P^{\prime}_{k|k - 1} = \lambda_{k} P_{k|k - 1} = S_{k|k - 1} S_{k|k - 1}^{T}$$

where $\lambda_{k}$ is the forgetting factor introduced at moment $k$. The measurement covariance matrix is:

10 $$\Lambda_{k} = P_{Y,k|k - 1} = \frac{1}{m}\sum\limits_{i = 1}^{2n} {\left( {Y_{k|k - 1}^{i} - \mathop {\hat{Y}_{k|k - 1} } } \right)\left( {Y_{k|k - 1}^{i} - \mathop {\hat{Y}_{k|k - 1} } } \right)}^{T} + R_{k - 1}$$

The measurement covariance matrix will increase when there is uncertainty in the tracking target.

11 $$\Lambda_{k + 1}{\prime} = \left\{ {\begin{array}{*{20}l} {\eta_{1} \eta_{1}^{T} ,} \hfill & {k = 0} \hfill \\ {\frac{{\rho *\Lambda_{k}{\prime} + \eta_{k + 1} \eta_{k + 1}^{T} }}{1 + \rho },} \hfill & {k \ge 1} \hfill \\ \end{array} } \right.$$

where $\eta_{k} = Y_{k} - \hat{Y}_{k|k - 1}$ is the measurement residual, $0 \le \rho \le 1$ is the weight coefficient determined as a result of the system data, and the relationship between $\Lambda_{k}{\prime}$ and $\Lambda_{k}$ is:

12 $$\Lambda_{k}{\prime} = \tau_{k} \Lambda_{k}$$

where $\Lambda_{k}{\prime} \tau_{k} = \max \{ 1,\frac{1}{m}tr(\Lambda_{k}{\prime} \Lambda_{k}^{ - 1} )\}$ is a scalar.

13 $$\lambda_{k} = \max \left\{ {1,\frac{{tr\left( {\Lambda_{k}{\prime} - R_{k} } \right)}}{{tr\left( {P_{Y,k|k - 1} - R_{k} } \right)}}} \right\}$$

The update step of the algorithm improved by the introduction of the adaptive forgetting factor is:

14 $$P_{k|k - 1} = S_{k|k - 1} S_{k|k - 1}^{T}$$

Covariance matrix after introduction of forgetting factor:

15 $$P_{k|k - 1}{\prime} = \lambda_{k} P_{k|k - 1} = S_{k|k - 1} S_{k|k - 1}^{T}$$

16 $$\chi_{k|k - 1}^{i} = \hat{X}_{k|k - 1} + S_{k|k - 1} \zeta_{i}$$

Covariance matrix:

17 $$P_{Y,k|k - 1} = \frac{1}{m}\sum\limits_{i = 1}^{2n} {Y_{k|k - 1}^{i} \left( {Y_{k|k - 1}^{i} } \right)^{T} } - \hat{Y}_{k|k - 1} \left( {\hat{Y}_{k|k - 1} } \right)^{T} + R_{k - 1}$$

18 $$P_{XY,k|k - 1} = \frac{1}{m}\sum\limits_{i = 1}^{2n} {\left( {\chi_{k|k - 1}^{i} - \hat{X}_{k|k - 1} } \right)\left( {Y_{k|k - 1}^{i} - \hat{Y}_{k|k - 1} } \right)}^{T}$$

Calculate the forgetting factor:

19 $$\Lambda_{k}{\prime} = \lambda_{k} P_{Y,k|k - 1}$$

Kalman gain:

20 $$K_{k} = P_{XY,k|k - 1} \left( {P_{Y,k|k - 1} } \right)^{ - 1}$$

Target status update:

21 $$\hat{X}_{k} = \hat{X}_{k|k - 1} + K_{k} \left( {Y_{k} - \hat{Y}_{k|k - 1} } \right)$$

State covariance matrix:

22 $$P_{k} = P_{k|k - 1} - K_{k} P_{Y,k|k - 1} K_{k}^{T}$$

The process noise covariance matrix Q and the measurement noise covariance matrix R in Kalman filtering are optimized by adjusting the parameters, which in turn update the time update and the measurement update of Kalman filtering.

## Case study

The algorithm is applied to an open pit iron mine for monitoring. Two monitoring points are selected in the mine, and optimized Kalman filter approach based on improved Gray Wolf algorithm (IGWO-OKF) and variance compensation adaptive Kalman filter are used to process the monitoring data of open pit deformation.

### Optimizing the Gray Wolf algorithm

The machine used in this design contains Intel Core i7 processor and the GPU is RTX 3060. in terms of software, matlab is used for simulation.

**Figure 1:**
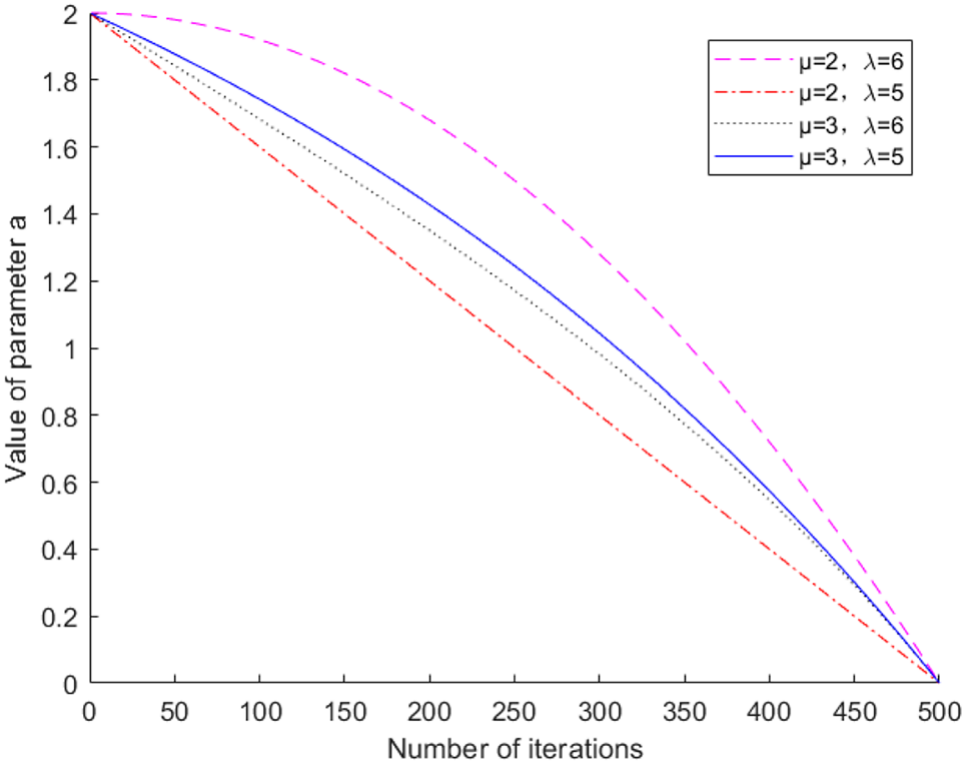
Variation curve of dynamic factors with different weights as a function of the number of iterations.

From Eq. (3), it can be known that the control parameter a is an important influence on the search parameter A, which directly affects the algorithm's local and global search ability. The four curves in Fig. 1 correspond to different parameter control strategies, which have different degrees of influence on the convergence speed of the algorithm and the solution results. From the analysis, the optimal regulation parameter, i.e., $\mu = 2,\lambda = 5$, is selected.

**Figure 2:**
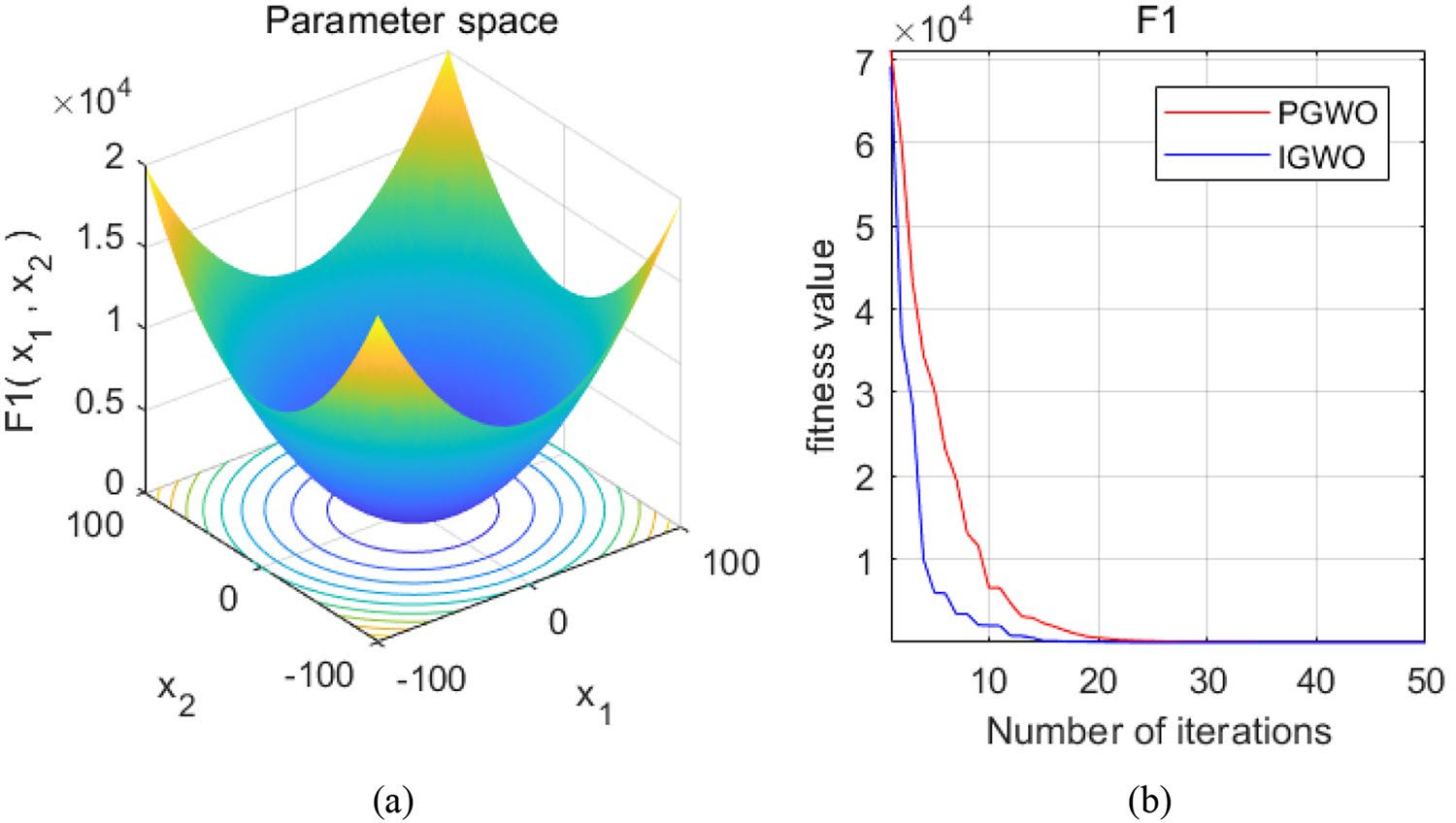
Graph of Gray Wolf iteration curves.

The optimal value of the objective function found by PGWO is 13.1649, and the optimal value of the objective function found by IGWO is 7.5488e-11. Therefore, the performance and effect of IWGO are better than that of PGWO. It can be seen from Fig. 2 that the number of iterations in the parameter space, IGWO and PWGO will gradually converge to a better solution with the increase of iterations, and IGWO will use fewer iterations, with faster convergence speed and better effect.

### IGWO optimized Kalman filtering

Variance Compensation Adaptive Kalman Filtering (VCAKF) is an improved Kalman filtering algorithm for nonlinear systems or systems with uncertainties. It improves the performance of the filter by adaptively estimating and compensating for the variance of measurement and process noise. In conventional Kalman filtering, the variance of the measurement and process noise is usually fixed, but in practical applications these may change over time or in the environment. Variance-compensated adaptive Kalman filtering better adapts to system dynamic changes and uncertainties by estimating and updating the noise variance in real time. Therefore, in this paper, the proposed improved Gray Wolf algorithm optimizes Kalman filtering with variance-compensated adaptive Kalman filtering to process the deformation monitoring data of an open pit mine, respectively.

**Figure 3:**
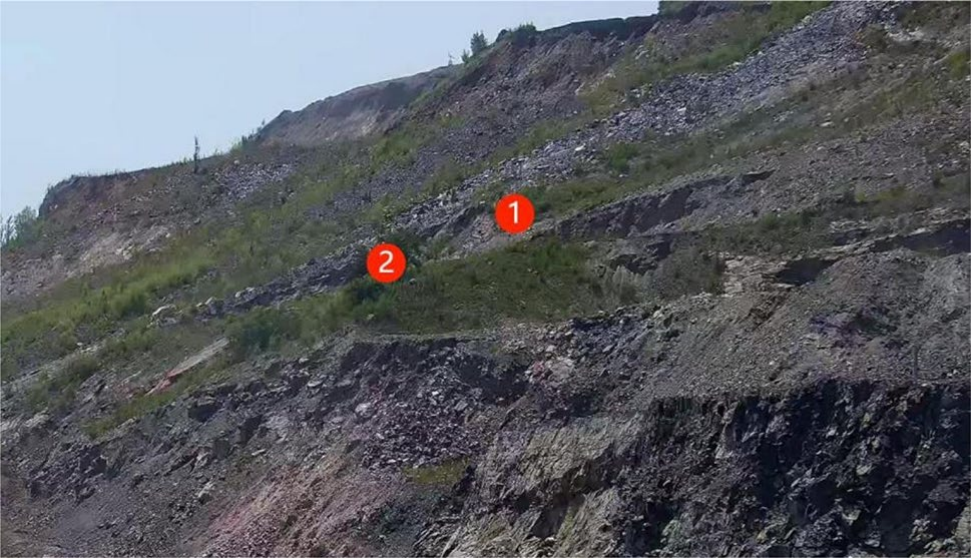
Layout of monitoring points.

According to the deformation data obtained by automatic deformation monitoring, the variance compensation adaptive Kalman filtering is applied to two deformation monitoring data on the hillside of an open-pit mine, and the filtered values are obtained. The data of the previous 9 periods are original data, and the data of the 10th period is predicted. Each period lasts 10 days. The results are compared with the actual observed values. The observation point is shown in Fig. 3.

By comparing the actual observed values of deformation monitoring point 1 with the filtered values in Table 1, it can be seen that the maximum residual error of the variance-compensated adaptive Kalman filter in the X-direction coordinate is −0.46 mm, and the minimum value of the X-direction coordinate residual error is 0.05 mm; in the Y-direction coordinate residual error has a maximum value of 0.49 mm, and its minimum value of the coordinate residual error is 0.07 mm.

**Table 1:** Raw data and variance-compensated adaptive Kalman data for monitoring site 1.

Phase	Measured value (m) X coordinate Y coordinate		Variance Compensation Adaptive Kalman (m) X coordinate Y coordinate		X offset/mm	Y offset/mm
1	514.7998	877.4571	514.7998	877.4571	0.00	0.00
2	514.7998	877.4559	514.7998	877.4560	0.05	0.07
3	514.7984	877.4556	514.7986	877.4557	0.22	0.18
4	514.7979	877.4552	514.7975	877.4555	−0.34	0.26
5	514.7950	877.4530	514.7953	877.4535	0.30	0.49
6	514.7963	877.4521	514.7967	877.4524	0.40	0.29
7	514.7952	877.4500	514.7948	877.4496	−0.43	−0.34
8	514.7939	877.4502	514.7935	877.4504	−0.44	0.24
9	514.7928	877.4491	514.7932	877.4496	0.38	0.46
10	514.7909	877.4460	514.7904	877.4459	−0.46	−0.11

As can be seen in Table 2, comparing the filtered values of the variance-compensated adaptive Kalman filter with the actual observed values, the maximum residuals of the coordinates in the X-direction of point 2 are found to be 0.49 mm; the maximum residuals of the coordinates in the Y-direction are found to be 0.46 mm; the maximum residuals of the filtered values are found to be no more than 0.5 mm in the variance-compensated adaptive Kalman filtering of point 2, and it can be seen that The stability of the variance compensated adaptive Kalman filter is relatively strong, and the residual values are relatively stable.

**Table 2:** Raw data and variance-compensated adaptive Kalman data for monitoring site 2.

Phase	Measured value (m) X coordinate Y coordinate		Variance Compensation Adaptive Kalman (m) X coordinate Y coordinate		X offset/mm	Y offset/mm
1	512.0429	877.0389	512.0429	877.0389	0.00	0.00
2	512.0430	877.0376	512.0430	877.0377	0.06	0.07
3	512.0411	877.0377	512.0414	877.0374	0.27	−0.27
4	512.0392	877.0357	512.0391	877.0360	−0.13	0.24
5	512.0377	877.0350	512.0375	877.0351	−0.22	0.06
6	512.0406	877.0342	512.0402	877.0347	−0.42	0.46
7	512.0368	877.0333	512.0369	877.0330	0.08	−0.26
8	512.0358	877.0324	512.0358	877.0324	−0.06	0.06
9	512.0337	877.0325	512.0339	877.0322	0.24	−0.32
10	512.0330	877.0303	512.0334	877.0307	0.49	0.40

Table 3, shows that comparing the actual observed value and the filtered value of the deformation monitoring point 1, it can be seen that the maximum residual value of the IGWO optimized Kalman filter in the X direction is 0.2 mm; the maximum residual value in the Y direction is 0.24 mm, and the difference of the coordinates can be seen that the IGWO optimized Kalman filter is closer to the real measurement value, and the range of error is obviously smaller, and the stability is enhanced.

**Table 3:** Monitoring site 1 raw data and IGWO optimized post-Kalman data.

Phase	Measured value (m) X coordinate Y coordinate		IGWO Optimization Kalman (m) X coordinate Y coordinate		X offset/mm	Y offset/mm
1	514.7998	877.4571	514.7998	877.4571	0.00	0.00
2	514.7998	877.4559	514.7998	877.4559	0.03	0.05
3	514.7984	877.4556	514.7985	877.4553	0.10	−0.24
4	514.7979	877.4552	514.7978	877.4553	−0.05	0.12
5	514.7950	877.4530	514.7949	877.4529	−0.02	−0.11
6	514.7963	877.4521	514.7963	877.4520	−0.03	−0.06
7	514.7952	877.4500	514.7951	877.4497	−0.10	−0.20
8	514.7939	877.4502	514.7938	877.4504	−0.13	0.24
9	514.7928	877.4491	514.7930	877.4493	0.20	0.12
10	514.7909	877.4460	514.7911	877.4459	0.15	−0.11

According to Table 4, comparing the filtered values of IGWO optimized Kalman filter with the actual observed values, it is found that the maximum residual value of the coordinates in the X direction of the No. 2 point is 0.25 mm; the maximum residual value of the coordinates in the Y direction is 0.22 mm, thus it can be seen that the stability of the IGWO optimized Kalman filter is relatively strong, and the residual value is relatively stable and has a good convergence.

**Table 4:** Monitoring site 2 raw data and IGWO optimized post-Kalman data.

Phase	Measured value (m) X coordinate Y coordinate		IGWO Optimization Kalman (m) X coordinate Y coordinate		X offset/mm	Y offset/mm
1	512.0429	877.0389	512.0429	877.0389	0.00	0.00
2	512.0430	877.0376	512.0430	877.0376	0.03	0.05
3	512.0411	877.0377	512.0412	877.0376	0.13	−0.11
4	512.0392	877.0357	512.0390	877.0358	−0.18	0.09
5	512.0377	877.0350	512.0377	877.0348	0.02	−0.19
6	512.0406	877.0342	512.0405	877.0341	−0.08	−0.11
7	512.0368	877.0333	512.0371	877.0334	0.25	0.08
8	512.0358	877.0324	512.0360	877.0325	0.15	0.13
9	512.0337	877.0325	512.0339	877.0327	0.23	0.22
10	512.0330	877.0303	512.0332	877.0303	0.25	0.01

**Figure 4:**
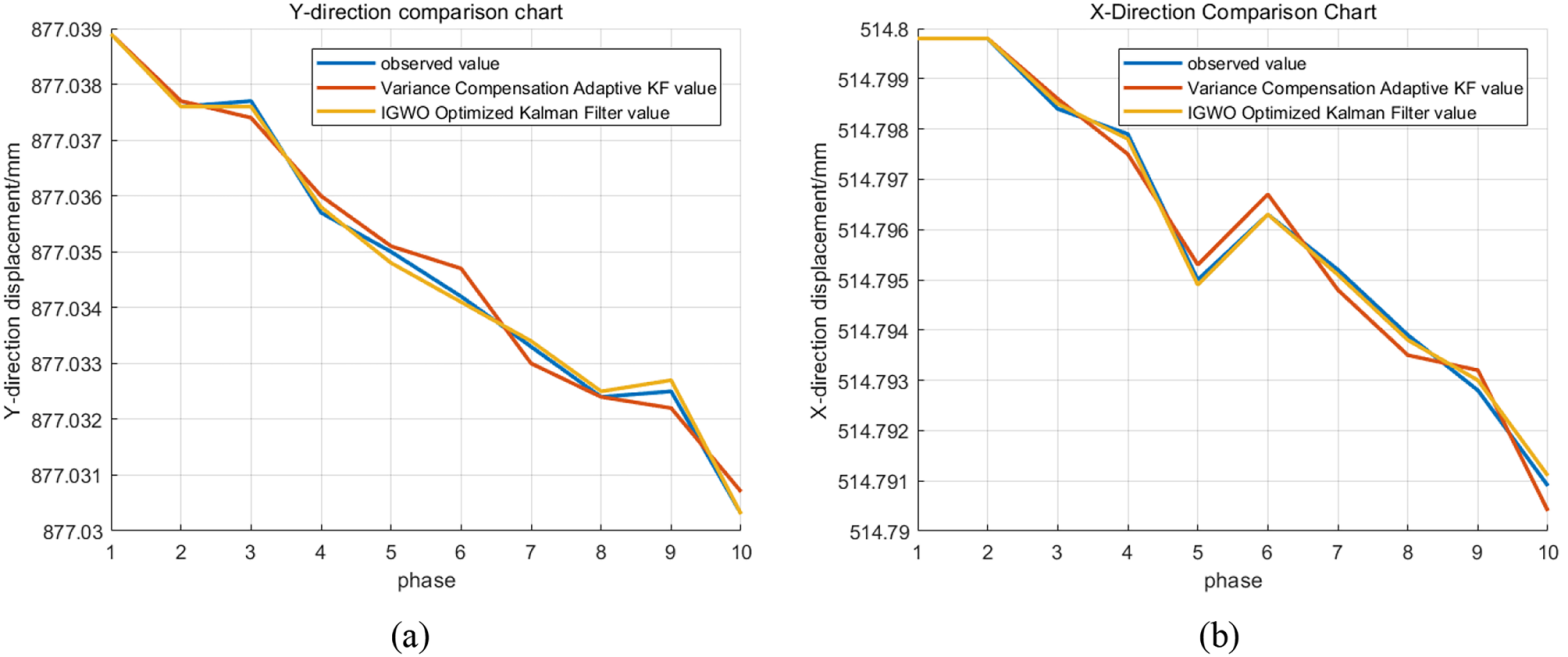
Displacement prediction map.

The following figure shows the measured and estimated data of the slope displacement trajectory. The predicted motion trajectory after simulation is shown in Fig. 4.

As shown in the figure the variance compensated adaptive Kalman filter starts to diverge in the fifth period, the proposed IGWO optimized Kalman filter has less filter fluctuation, more stable filter values and higher robustness than the variance compensated adaptive Kalman filter.

## Conclusion

In order to improve the accuracy of landslide monitoring, a IGWO-optimized Kalman filter is proposed to optimize the covariance matrix of process noise and measurement noise in the Kalman filter using the improved Gray Wolf algorithm. The experimental results demonstrate that the use of the IGWO-optimized Kalman filter model achieves faster convergence to the actual observations and less volatility than the traditional variance-compensated adaptive Kalman filter. This indicates that the model has stronger convergence ability, better stability, and better filtering results and deformation prediction accuracy. In contrast, the variance-compensated adaptive Kalman filtering algorithm is inferior to the IGWO-optimized Kalman filtering model in terms of filtering effect and prediction, and its accuracy, consistency, and robustness are all lacking. The proposed filtering model can be applied in landslide monitoring, and in the future, it is planned to enhance the improvement on the measurement update and further optimize the Kalman filter.

## Data Availability

The datasets generated and/or analysed during the current study are not publicly available due to privacy issues. If sosmeone wants to obtain data from this study, please contact Zheming Pang.
